# Enhanced ICP for the Registration of Large-Scale 3D Environment Models: An Experimental Study

**DOI:** 10.3390/s16020228

**Published:** 2016-02-15

**Authors:** Jianda Han, Peng Yin, Yuqing He, Feng Gu

**Affiliations:** 1 State Key Laboratory of Robotics, Shenyang Institute of Automation, Chinese Academy of Sciences, Shenyang 110016, China; jdhan@sia.cn (J.H.); fenggu@sia.cn (F.G.); 2 University of Chinese Academy of Sciences, Beijing 100049, China

**Keywords:** 3D model registration, multiple heterogeneous mobile robots, enhanced ICP

## Abstract

One of the main applications of mobile robots is the large-scale perception of the outdoor environment. One of the main challenges of this application is fusing environmental data obtained by multiple robots, especially heterogeneous robots. This paper proposes an enhanced iterative closest point (ICP) method for the fast and accurate registration of 3D environmental models. First, a hierarchical searching scheme is combined with the octree-based ICP algorithm. Second, an early-warning mechanism is used to perceive the local minimum problem. Third, a heuristic escape scheme based on sampled potential transformation vectors is used to avoid local minima and achieve optimal registration. Experiments involving one unmanned aerial vehicle and one unmanned surface vehicle were conducted to verify the proposed technique. The experimental results were compared with those of normal ICP registration algorithms to demonstrate the superior performance of the proposed method.

## 1. Introduction

One of the main applications of mobile robots is large-scale perception of the outdoor environment. Recently, many studies have focused on fusing environmental data from multiple robots, especially heterogeneous robots such as unmanned aerial vehicles (UAVs), unmanned ground vehicles (UGVs), unmanned surface vehicles (USVs), and even remoted operated vehicles (ROVs) to achieve better and complementary perception. However, the huge differences in experimental data obtained by heterogeneous robots, such as in the view angles and resolution, make combining the data difficult, especially with the demand for highly accurate perception.

The data fusion technique of 3D model registration (3D-MR) is extensively used in medical image registration [1,2], simultaneous localization and mapping (SLAM) of mobile robots [3,4,5], remote sensing and image processing [6], *etc.* Existing 3D-MR algorithms may be categorized into two classes: Featured-Based and featureless. Feature-based 3D-MR predefines some offline descriptors, such as Harris corners [7], Susan corners [8], and spin-images [9]. These descriptors are then used as features to find correspondences between the two 3D point clouds that need to be fused. Of these descriptors, the spin-image has been proven to be accurate and robust [10,11,12]. However, approaches that use it suffer from a heavy computational burden [10]. Moreover, the performance of feature-based MR depends on the accuracy of the preselected descriptors. This may limit its applicability to large-scale outdoor environment registration, for which accurate descriptors may be impossible to obtain. In order to reduce the feature dependency, Bao *et al.* [13] proposed using semantic prior information for dense object reconstruction. Ho and Gibbins used semantic features to align city-scale LiDAR point clouds [14].

In contrast to feature-based MR, the featureless scheme can be used to model unstructured environments where accurate features are difficult to predefine [14]. Iterative closest point (ICP) [15,16,17] is one of the most commonly used featureless 3D-MR algorithms. With ICP, one point cloud (normally called the reference or target) is fixed, while the other (called the source) is transformed to match the reference. The algorithm iteratively revises the transformation to minimize the distance between the two point clouds. Point-to-point ICP calculates the distance between two paired points and optimizes the distance by gradient descent. Thus, its performance closely depends on a good initial estimate. On the other hand, point-to-plane ICP takes advantage of the surface normal information to improve the robustness to the initial estimate. Plane-to-plane ICP uses the surface structure to measure the distance and has been proved to be more robust with respect to a large initial transformation error [18].

ICP algorithms have also been applied to take advantages of multi-resolution data. For example, Jost *et al.* [19,20] used multi-resolution ICP (M-ICP) to accelerate the registration procedure by scattering the point cloud at a lower resolution level. This scheme can also improve the robustness against the initial estimation. However, when used for large-scale outdoor environment model registration, most ICP algorithms may suffer from the local minimum problem. This is mainly due to the gradient-descent-based optimization procedure, which cannot guarantee a global optimal resolution. A normal approach to resolving the local minimum problem is deliberately selecting the initial estimation so that the iterative calculation will completely avoid local minima. However, how to guarantee that the initial value is sufficiently accurate is still an open problem, especially when the data are from different view angles and have different resolutions. Most recently, Yang *et al.* [21] proposed global optimal ICP (GO-ICP) to solve the local minimum problem. GO-ICP combines the ICP framework with a branch-and-bound (BnB) scheme to try to search the space more efficiently and thus guarantee global optimization. However, the high computational burden may be a problem when GO-ICP is used for the model registration of a large-scale outdoor environment, where unstructured datasets may involve large amounts of sensory data.

In this paper, we propose an enhanced ICP algorithm for the fusion of cloud points obtained by heterogeneous robots. Three enhancements are presented: (1) a hierarchical searching scheme that is combined with the octree-based 3D modeling technique to improve the robustness with respect to the initial modeling error and realize coarse-to-fine registration of large-scale multi-resolution data; (2) an early warning mechanism to perceive the local minimum problem; and (3) a heuristic escape scheme based on sampling potential transformation vectors to avoid local minima. Experiments using one UAV and one USV, both carrying cameras onboard, to measure a riverside environment were conducted to verify the proposed technique. The contents of this paper are organized as follows: first, the ICP algorithms are introduced in Section 2. Then, the proposed enhancement techniques are explained in Section 3. In Section 4, the experimental setup is introduced, and an analysis of the results along with a comparison with the results of normal ICP algorithms is presented for an evaluation of the performance of the proposed method. Finally, the conclusions and future work are discussed in Section 5.

## 2. Preliminaries

Let *P ∈ {R^N^}* and *Q ∈ {R^M^}* represent the 3D datasets of the scan and global models, respectively, where *N* and *M* are the point numbers inside *P* and *Q*, respectively. Without losing generality, if *M* > *N*, the standard ICP is to find the sub-point sets *{q_i_}^N^_i=1_* in model *Q* that are most similar to the scan model *P*, *i.e.*, to solve the problem:

(1) $$\begin{array}{l} \underset{T}{\min}(\sum_{i}^{N}||(Tp_{i}-q_{i}|{|}^{2})\\ T=\left[\begin{array}{cc} R & t\\ 0 & 1 \end{array}\right]\\ s.t.\;R^{T}R=I,\det(R)=1 \end{array}$$

where *T ∈ T^4 × 4^* is the combination of the rotation matrix *R* and translation vector *t*. Thus, the registration problem of two 3D models is converted into an optimization problem. However, the standard ICP cannot guarantee an optimal match and may suffer from local minima when a bad initial registration is used. Furthermore, the ICP itself cannot indicate whether or not it has been trapped into a local minimum.

Generalized-ICP (G-ICP) [18] uses the plane-to-plane scheme to improve the robustness. In G-ICP, all of the points in *P* and *Q* can be remodeled as a Gaussian distribution:

(2) $$\begin{array}{l} p_{i}\sim N(p_{i}^{m},C_{i}^{P})\\ q_{i}\sim N(q_{i}^{m},C_{i}^{Q}) \end{array}$$

where *p^m^_i_* and *q^m^_i_* are the measured points and *{C^P^_i_}* and *{C^Q^_i_}* are the covariance matrices associated with the measured points. Usually, *p_i_* and *q_i_* are assumed to be independent of each other.

For the transformation *T*, a new transformation error for *p_i_* and *q_i_* can be defined as:

(3) $$d_{i}^{\left(T\right)}=q_{i}-Tp_{i}$$

Thus, *d^(T)^_i_* is also a stochastic variant with the following Gaussian distribution:

(4) $$d_{i}^{\left(T^{*}\right)}\sim N({\hat{q}}_{i}-T^{*}{\hat{p}}_{i},C_{i}^{Q}+T^{*}C_{i}^{P}{\left(T^{*}\right)}^{T})$$

By using the maximum likelihood estimate, the ICP in Equation (1) can be transformed into a probabilistic model:

(5) $$\begin{array}{l} T=\underset{T}{\arg\max}\prod_{i}p(d_{i}^{\left(T\right)})\\ \;=\underset{T}{\arg\max}\sum_{i}\log(p(d_{i}^{\left(T\right)}))\\ \;=\underset{T}{\arg\min}\sum_{i}{\left(d_{i}^{\left(T\right)}\right)}^{T}{\left(C_{i}^{Q}+TC_{i}^{P}T^{T}\right)}^{-1}d_{i}^{\left(T\right)} \end{array}$$

If we set *C^Q^_i_* = *I* and *C^p^_i_* = 0, the above equation can be converted to the original ICP form:

(6) $$\begin{array}{l} T=\underset{T}{\arg\min}\sum_{i}{\left(d_{i}^{\left(T\right)}\right)}^{T}d_{i}^{\left(T\right)}\\ \;=\underset{T}{\arg\min}\sum_{i}||Tp_{i}-q_{i}|{|}^{2} \end{array}$$

G-ICP computes the covariance matrices along the direction normal to the local surface of each point, and the searching regions are larger compared with that of the standard point-to-point ICP. Thus, the possibility of G-ICP falling into a local minimum is reduced, and the robustness against measurement noise is improved. However, G-ICP increases the computational burden because of the stochastic calculations.

## 3. New Proposed Registration Algorithm

**Figure 1:**
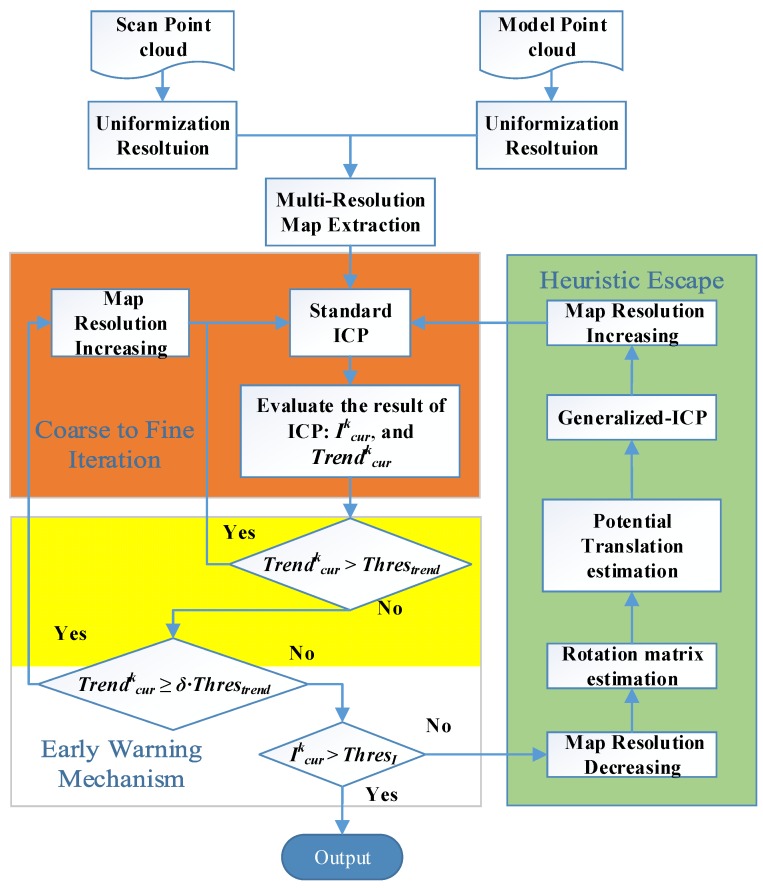
Pipe flow of the enhanced ICP: Coarse-to-Fine Iteration works as a Multi-Resolution ICP registration step; Early Warning Mechanism is introduced to estimate the potential local optima; Heuristic Escaping help the data point cloud escape from the current local optimal by estimating the potential optimal transformation.

Figure 1 shows a flowchart of our new proposed algorithm. It includes the following four steps:

*Step I.*  Point cloud standardization and extraction

Transform both the scan point cloud *P* (*i.e.*, local 3D model with *N_P_* points) and model point cloud *Q* (*i.e.*, global 3D model with *N_Q_* points) to the same resolution level through the use of the OctoMap [22] data structure. All different resolution level of a single point cloud can be generated from the same OctoMap.

*Step II.*  Coarse to fine iteration
  (a) Align the scan point cloud *P* and model point cloud *Q* at the current resolution level.
  (b) Calculate the efficiency of the current ICP registration by using the registration index *I^k^_cur_* and tendency index *Trend^k^_cur_*, where *k* represents the *k*th resolution level and *cur* represents the current ICP registration.
*Step III.*  Early warning mechanism
  (a) Adjust the resolution level based on the value of *Trend^k^_cur_*.
  (b) If *Trend^k^_cur_* is bigger than a given positive threshold, go directly to Step II. If *Trend^k^_cur_* is just bigger than zero, update the resolution to a higher level and then go to Step II.
  (c) If *I^k^_cur_* is bigger than the given threshold, the algorithm has found a global optimal result, and go to Step V. Otherwise, the early warning has been triggered, and go to Step IV.
*Step IV.*  Heuristic escape
  (a) Cluster the current aligned scan point cloud *P* based on distances between each point in *P* with its closest point in the model point cloud *Q*. Then, extract the biggest clustered point cloud *P_merge_* with the distance below the given threshold *Thres_cluster_*.
  (b) Estimate the normal vector and normal surface of the point cloud *P_merge_* and transform the current scan point cloud into six temporary scan point clouds *P^(n)^_temp_*, where n is from 1 to 6;
  (c) Weight each transformation vectors according to the registration index at each temporary scan point cloud *P^(n)^_temp_* and generate the transformation vector according to the ICP registration.
  (d) Estimate the potential translation *T_escape_* based on the weighted translation vectors and then transform the scan point cloud *P* according to the estimated translation vector. Go to Step III.

### 3.1. Octree-Based 3D Map Extraction

3D point cloud models obtained by using distinct devices or different platforms usually differ in scale, noise, and especially resolution. We used OctoMap to unify the point clouds with different resolution levels and generate multi-resolution maps based on the hierarchical octree data structure.

According to Hornung *et al.* [22], the occupancy of each OctoMap lead node or the highest-resolution map is updated according to the observations {*s*1:*i*} and the initial occupancy estimation:

(7) $$L{\left(n|s_{1:t}\right)}^{0}=L{\left(n|s_{1:t-1}\right)}^{0}+L{\left(n|s_{t}\right)}^{0}$$

where *L(n|s_1:t_)*^0^ represents the *n*th leaf node of OctoMap and *L(n|s_t_)*^0^ is the log-odds measurement of the *n*th leaf node based on current observations. The lower-resolution level node is generated directly from a higher-resolution level node:

(8) $$L{\left(m|s_{1:t}\right)}^{i+1}=\sum_{j}^{8}L{\left(n_{j}|s_{1:t}\right)}^{i}$$

where *L(m|s_1:t_)^I+1^* refers to eight lower-resolution nodes *L(m|s_1:t_)^I+1^* because of the 3D octree structure [23]. Thus, multi-resolution maps can be generated from and saved in a single OctoMap structure.

### 3.2. Early Warning Mechanism

Because traditional ICP methods cannot tell whether or not they have been trapped in a local minimum, we introduced the early warning mechanism to perceive the local minimum situation. We defined the registration index *I^k^_cur_* for the current resolution level *k*:

(9) $$\begin{array}{l} I_{cur}^{k}=\exp(1/D_{k})\\ D_{k}=(\sum_{i=1}^{N_{p}}||q_{i}^{k}-T_{opt}p_{i}^{k}|{|}^{2}) \end{array}$$

where *D_k_* is the sum of the Euclidean distances between the scan point *p^k^_i_* and its nearest model point *q^k^_i_*. *T_opt_* is the optimal transformation matrix based on the current alignment. Actually, the registration index *I^k^_cur_* defines the registration error on the *k*th resolution level. If the registration index is beyond the given error *Thres_I_*, the current registration has meet the error tolerating band.

However, a single registration index cannot be used to perceive the local minimum situation. We defined the tendency index *Trend^k^_cur_* to describe the tendency of the current *k*th resolution level’s registration procedure:

(10) $$Trend_{cur}^{k}=\frac{\left(I_{cur}^{k}-I_{pre}^{k}\right)}{T_{cur}^{k}}$$

where *I^k^_pre_* is the previous registration index on the *k*th resolution level and *I^k^_cur_* is the current registration index on the *k*th resolution level. *T^k^_cur_* is the computation time of the current registration loop on the *k*th resolution level. The tendency index *Trend^k^_cur_* measures the velocity of the current registration procedure at the *k*th resolution. Usually, a higher velocity means an efficient local optimal, while a lower or in some cases even negative velocity may mean the registration procedure has reached a local minimum situation. The resolution level *level_re_* is determined by the tendency index *Trend^k^_cur_*:

(11) $$level_{re}=\left\{ \begin{array}{l} level_{re},Trend_{cur}^{k}>Thres_{trend}\\ \frac{level_{re}}{2},Thres_{tend}\geq Trend_{cur}^{k}\geq \delta \cdot Thres_{trend}\\ level_{re}\cdot 2,Trend_{cur}^{k}<\delta \cdot Thres_{trend} \end{array}\right.$$

where *δ* is a constant parameter between 0 and 1. If the tendency index is beyond the given threshold *Thres_Trend_*, the registration may achieve more accurate results based on the current resolution level. Then, the coarse-to-fine registration scheme can continue. However, if this index is only bigger than *δ* times *Thres_Tend_*, the registration process may not have been able to achieve efficient improvement at the current resolution level. Then, both point clouds are transformed into a higher resolution level through the use of OctoMap, and the registration process continues. Otherwise, the registration process has hit a local optimum. Then the optimum is determined to be a local minimum or global optimum according to the registration index *I^k^_cur_*. If the registration index is beyond *Thres_I_*, the registration process ends, and the aligned point clouds are output. Otherwise, the current registration may be a local minimum. The early warning is triggered, and the algorithm enters the heuristic escape scheme.

### 3.3. Heuristic Escape

The heuristic escape scheme helps the scan point cloud exit the local minimum by estimating the proper rotation angle *R_escape_* and translation *T_escape_* based on sampled potential rotation angles and transformation vectors. Both rotation angles and translation vectors are relayed to the merged scan and model point clouds.

To extract the merged point cloud, the distance between each point in the scan point cloud *P* and the closest point in the model point cloud *Q* is evaluated, and the scan points with a distance greater than the constant threshold *Thres_cluster_* are filtered out. The remaining points in the scan point cloud are clustered by the k-means method, and the biggest clustered point cloud *P_merge_* is extracted as the merged point cloud. According to Rusu *et al.* [24], the normal vector of *P_merge_* can be estimated from the eigenvalue of the covariance matrix of the point cloud:

(12) $$\begin{array}{l} Cov=\frac{1}{N}\sum_{i=1}^{N}\left(p_{i}-\bar{p}\right)\cdot {\left(p_{i}-\bar{p}\right)}^{T}\\ Cov\cdot v_{j}=\lambda_{j}\cdot v_{j},j\in \{1,2,3\}\\ \bar{p}=\frac{1}{N}\sum_{i=1}^{N}p_{i} \end{array}$$

where *N* is the number of points in the point cloud, *λ_j_* is the *j*th eigenvalue of the matrix *Cov*, *v_t_* is its corresponding eigenvector, and *j* is from 1 to 3. We assumed the eigenvalue is ordered by *λ_1_* ≥ *λ_2_* ≥ *λ_3_*, so *v_3_* can be taken as the normal vector of the point cloud *P_merge_*. Then, the normal surface of *P_merge_* can also be determined by *v_3_*.

Based on the current position, we can measure the registration index for six equally divided rotation angles on the normal surface. The escape rotation *R_escape_* is the rotation matrix *R_j_* of the relative angle *j* with the highest registration index:

(13) $$R_{escape}=\{R_{j}|j=\underset{i=0,60,120...300}{\max}I_{i}\}$$

where *I_i_* is the registration index of the corresponding rotation angle *i* in degree.

**Figure 2:**
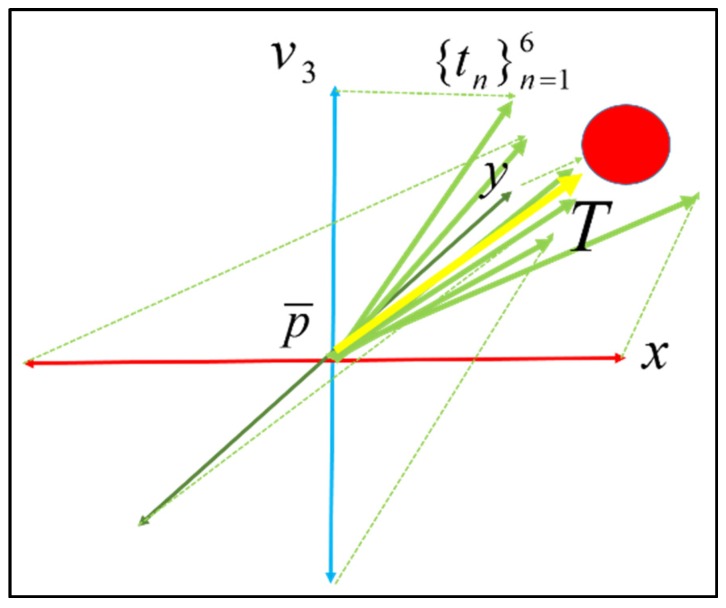
Escape direction estimation: *v_3_* is the normal vector of the point cloud *P_merge_*, the potential initial transformations are allow the 3 axis of *x*, *y* and *v_3_*. Dotted green lines are the transformations estimated by ICP registration and the thick green lines *t_n_* are the combination of initial transformation and ICP estimation. Thick yellow line represent of the combination of *t_n_*, and red ball is desired registration.

To estimate the escape translation, the scan point cloud is transformed to temporary scan point clouds *P^(n)^_temp_* along the six transformation matrices *T^potential^_n_*. Two matrixes are along the normal vector (*i.e.*, blue axis), and the other four are from the normal surface *P_merge_* (*i.e.*, red and green axes), where *n* is from 1 to 6. Then, each temporary scan point cloud is aligned to the model point cloud by the standard ICP method. The corresponding potential transformation vector *t_n_* is generated by

(14) $$t_{n}=\left[\begin{array}{cc} 0_{3\times 3} & 1_{3\times 1} \end{array}\right]T_{n}^{ICP}\cdot T_{n}^{pote\text{ntial}}\left[\begin{array}{c} 1_{3\times 1}\\ 0 \end{array}\right]$$

where *T^ICP^_n_* is the relative ICP registration matrix. Figure 2 shows the six potential transformation vectors as the green lines. Then, *t_n_* is weighted to the registration index of *P^(n)^_temp_*.

Finally, the proper escape translation *T_escape_* can be estimated based on the six weighted potential transformation vectors:

(15) $$\begin{array}{l} T_{escape}=\frac{1}{6}\sum_{n=1}^{6}\theta_{n}t_{n}\\ \theta_{n}=\frac{I_{n}}{\sum_{n=1}^{6}I_{n}},n=1,2,3...6 \end{array}$$

where *θ_n_* is the normalized weight of the *k*th potential transformation. The escape translation is shown as the yellow line in Figure 2. Then, the scan point cloud is transformed according to the escape translation. Simultaneously, the resolution level of the point clouds is transformed to a higher resolution level, and the coarse-to-fine iteration scheme is continued.

## 4. Experiments and Results

### 4.1. Experiment Setup

**Figure 3:**
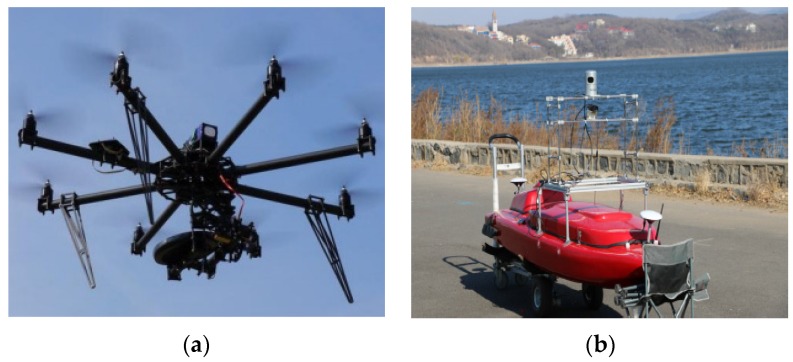
Experiment Vehicles: (**a**) a UAV is equipped with a Velodyne VLP-16 LiDAR to generate the Model point cloud, where the resolution level is 1 m; (**b**) a USV is equipped with a Velodyne 32e Lidar, which could generate the local Scan point cloud at the resolution level at 0.2 m.

To verify the performance improvement of the proposed algorithm, experiments were conducted on the cooperation of multiple heterogeneous UAV and USV at a river bank. Both robots were equipped with a navigation system for pose measurement and LiDAR for environment perception, as shown in Figure 3. The pose of each platform was estimated with the inertial measurement unit (IMU) and differential Global Positioning System (GPS) at an output frequency of 100 Hz.

As shown in Figure 3a,b, the model was extracted from a bay. The points were collected by a Velodyne VLP-16 LiDAR mounted on the UAV that generated 300,000 points per second. The model point cloud was generated through an offline SLAM method. The scan point cloud was collected by a Velodyne 32e LiDAR mounted on USV that generated about 700,000 points per second. In our experiments, all of the environmental data were gathered online on an embedded board, and the registration algorithm ran offline on a laptop (Think-pad x220: Intel i5-2410 m 4 core @ 2.30 GHz CPU and 8 GB RAM) from Lenovo. The software was programmed in C++ with the Robot Operating System (ROS) [25] framework and Point Cloud Library (PCL) [26].

The small initial translation error was [10 m, 15 m, 10 m] on the roll, pitch, and yaw axes, and the large initial translation error was about 20 m. The small initial rotation error was about [5.7°, 5.7°, 15.6°], and the large initial rotational error was about 30°.

**Figure 4:**
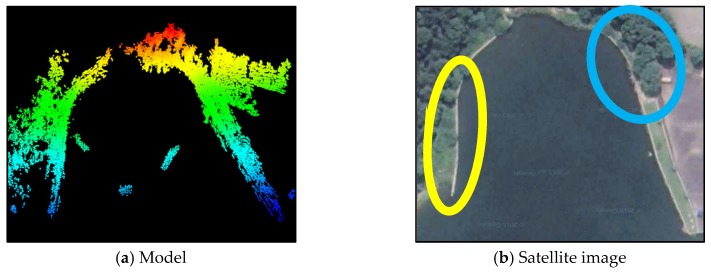
Experimental site (middle) and its 3D models: (**a**) 3D model obtained from USV; (**b**) satellite imagery of the experimental site, the green circled area is the slender bank and the blue circled area is the triangular area.

We tested the registration methods in two different registration experiments: A slender bank with complex terrain, which is circled in yellow in Figure 4b; and a triangular area with diverse elevations, which is circled in blue in the same figure. Table 1 gives the details of each experiment. To verify the robustness against different resolution levels, the model point clouds were kept at the same resolution level in both experiments, but the resolution level of the scan point clouds was set to 0.5 m for the slender bank and 0.2 m for the triangular area.

**Table 1:** Experimental conditions.

No.	Datasets	Number of Points	Resolution
Slender bank			
ExI	Model	212,912	1 m
	Scan	40,300	0.5 m
Triangular area			
ExII	Model	212,912	1 m
	Scan	125,493	0.2 m

To quantitatively evaluate the registration accuracy, the following registration error index Δ*T* was defined:

(16) $$\Delta T=\left[\begin{array}{cc} \Delta R & \Delta t\\ 0 & 1 \end{array}\right]=T_{r}\times T_{e}$$

where *T_r_* is the corresponding transformation obtained through using different registration algorithms and *T_e_* is the initial transformation error.

Similarly, the translation error *e_t_* and rotation error *e_r_* can be defined based on the Euclidean norm of the translation vector and geodesic distance as follows:

(17) $$\begin{array}{l} e_{t}=||\Delta t||\\ e_{r}=\arccos(\frac{trace(\Delta R)-1}{2}) \end{array}$$

The followed subsections present detailed analysis of the experiment results to demonstrate the improvement in performance.

### 4.2. Multi-Resolution Map Generation

**Figure 5:**
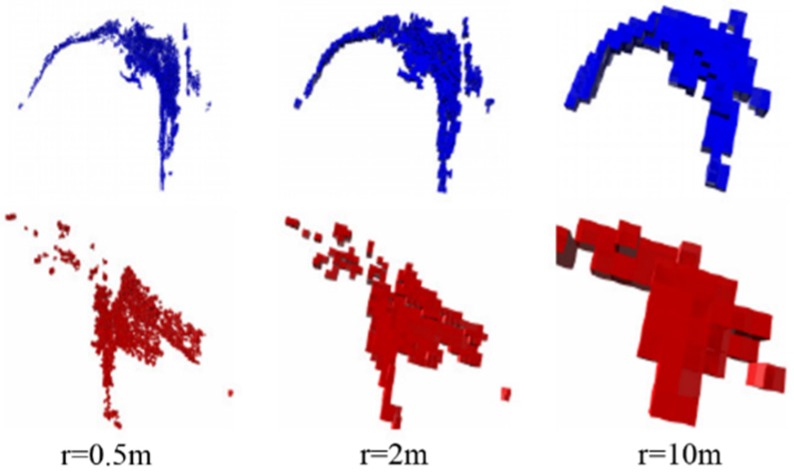
Multi-resolution maps, where *r* is the resolution of the 3D model. The blue ones are the global model maps, and the red ones are the local scan maps, three different resolution levels (0.5 m, 2 m and 10 m) are listed in this figure.

Figure 5 shows both scan and model point clouds at resolution levels of 0.5, 2, and 10 m. With the hierarchical property of the OctoMap structure [22], all different resolution-level maps of the same point cloud could be generated from a single OctoMap data structure. The lower-resolution level map could be directly generated from a higher-resolution level map, as shown in Equation (8).

At the 10-m resolution level, the maps were not sensitive to noise and outliers. Everything was transformed into a simple data structure. This property improved the robustness against the initial registration error.

### 4.3. Warning and Escape

**Figure 6:**
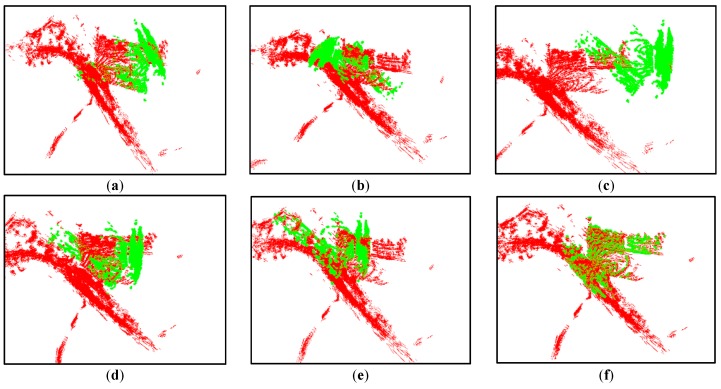
Local minima of the triangular area test: (**a**–**e**) show five local minimum registrations in the triangle area test; (**f**) shows the desired registration result.

**Figure 7:**
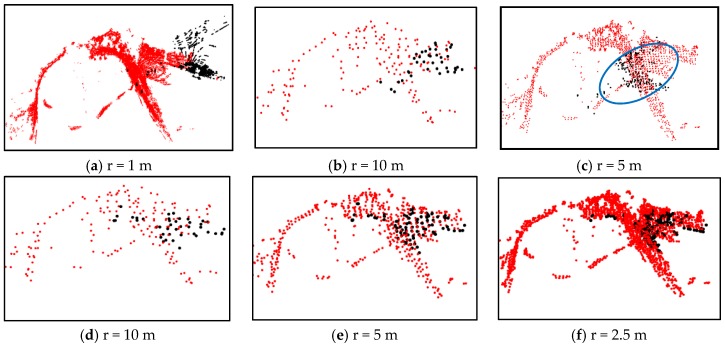
Heuristic escape: (**a**) shows the original transformation; (**b**–**f**) show the multi-resolution registration with the heuristic escape; the registration process hits a local optimal in the blue circle of (**c**); (**d**) shows the heuristic escaping transformation; (**e**–**f**) shows the following registration process.

We then verified the robustness of our proposed heuristic escape scheme against the local minimum problem. Figure 6a–e lists five local minimum registrations without our heuristic escape, Figure 6f presents one global optimal registration with our escape scheme. Figure 7 gives a detailed registration process for the ExII triangular area case with an initial translation error of [10 m, 30 m, 10 m] and rotation error of [−10°, 10°, 55°]. Figure 7a shows the initial point clouds of both scan (colored in black) and model (colored in red). Figure 7b,c shows the coarse-to-fine registration scheme. At the resolution level of 5 m, as shown by the blue circle of Figure 7c, the tendency index *Trend^k^_cur_* according to Equation (10) was below the expected δ times *Thres_Tend_*. The registration index *I^k^_cur_* evaluated by Equation (9) was also lower than *Thres_I_*. Thus, the early warning was triggered, and the registration process entered the heuristic escape step. The resolution level was lowered to 10 m, as shown in Figure 7d, and a new transformation was generated by the heuristic escape estimation based on Equations (9)–(11). Finally, the registration result reached a global optimum, as shown in Figure 7f.

**Figure 8:**
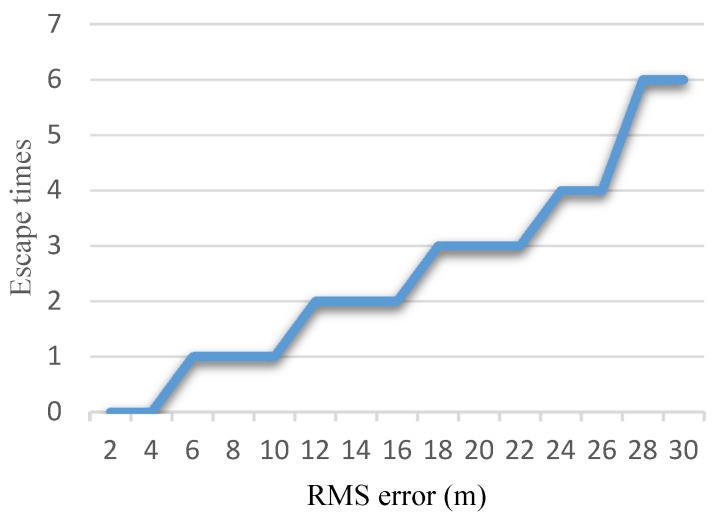
Escape times *vs.* RMS error.

Different initial transformation errors led to variations in the escape times. Figure 8 shows the relation between the root mean square (RMS) of the initial transformation error and the escape times. As the RMS error increased, the escape time grew with a ladder pattern.

### 4.4. Convergence

**Figure 9:**
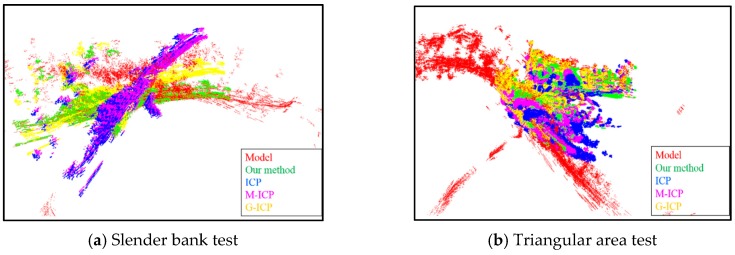
Registration results for the normal initial error test. In the slender bank test, the translation error was [5, 4, 4], and the rotation error was [17.1°, 20°, 34.2°], our proposed method make the registration with the RMS error at 1.1 m and rotation error at 1.5°. In the triangular area test, the translation error was [4, 3, 10], and the rotation error was [10°, 20°, 34.2°], our proposed method guarantee the registration result with the RMS error at 0.4 m and rotation error at 1.1°.

**Figure 10:**
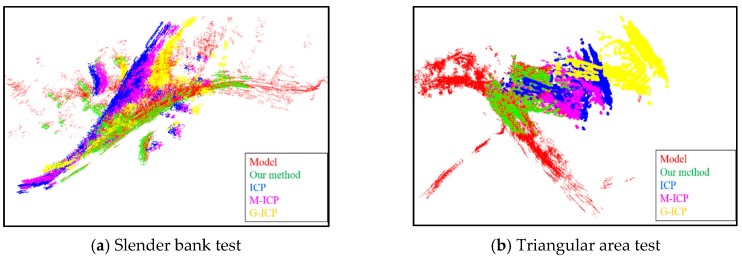
Registration results of the abrupt turn test. In the slender bank test, the translation error was [15, 14, 20], and the rotation error was [17°, 20°, 190°], our proposed method make the registration with the RMS error at 1.5 m and rotation error at 2.3°. In the triangular area test, the translation error was [14, 15, 20], and the rotation error was [10°, 20°, 171°], our proposed method guarantee the registration result with the RMS error at 2.3 m and rotation error at 1.7°.

Besides our proposed enhanced ICP, we also evaluated three other ICP-based methods for comparison: Standard-ICP, M-ICP, and G-ICP. Figure 9 and Figure 10 show the results for the slender bank and triangular area experiments. The red point cloud represents the model point cloud *Q*. The green, yellow, purple, and pink point clouds represent the registration results of the enhanced ICP, standard-ICP, M-ICP, and G-ICP, respectively. Each method was tested for both the normal initial transformation error and abrupt turn case in the experiment.

For the normal initial transformation error case, the initial translation error was randomly sampled within [±10, ±10, ±10], and the rotation error was sampled within [±20°, ±20°, ±40°]. Although all methods obtained acceptable results for the triangular area test, as shown in Figure 9b, the other methods failed to match the model point cloud for the slender bank test owing to the complexity of the terrain as shown in Figure 9a. In contrast, our proposed enhanced ICP method guaranteed correct results with a final RMS error of 1.1 m and rotation error of 1.5°. Table 2 summarizes the registration results of 40 normal initial transformation error tests on the slender bank and triangular area in Detail 2. On average, our proposed method could achieve an accurate match even with a rough initial error.

To verify the abrupt turn problem with the different ICP-based methods, we set the initial rotation error on the *z*-axis around 160°–220°, as shown in Figure 10. The Standard ICP, M-ICP, and G-ICP became trapped in a local minimum in both the slender bank and triangular area test. Our proposed enhanced ICP could efficiently estimate the local minimum problem by using the early warning mechanism and eventually escape with a proper transformation by using our heuristic escape scheme.

**Table 2:** Registration results with a normal initial error.

Method	Translation Error *e_t_*			Rotation Error *e_r_*		
	min	μ_t_	σ_t_	min	μ_r_	σ_r_
Slender Bank Experiment						
ICP	1.45 m	8.34 m	6.32 m	3.4°	24.1°	10.2°
M-ICP	1.55 m	6.57 m	4.31 m	1.6°	15.8°	5.9°
G-ICP	0.45 m	5.11 m	2.31 m	0.2°	12.09°	3.8°
OUR	0.23 m	1.78 m	1.98 m	0.4°	1.7°	1.2°
Triangular area Experiment						
ICP	1.24 m	8.23 m	9.21 m	2.3°	19.4°	12.2°
M-ICP	1.77 m	5.34 m	6.53 m	2.1°	15.4°	8.6°
G-ICP	0.30 m	4.23 m	3.34 m	0.5°	12.7°	6.1°
OUR	0.10 m	1.32 m	1.42 m	1.1°	2.8°	1.8°

*μ_t_* is the average translation error; *σ_t_* is the variance of the translation error; *μ_r_* is the average rotation error; and *σ_t_* is the variance of the rotation error. The translation errors were within [±10, ±10, ±10], and the rotation errors were within [±20°, ±20°, ±40°].

Table 3 summarizes the results of 40 abrupt turn tests in detail. Even with a tough abrupt turn, our proposed enhanced ICP method was able to guarantee matching accuracy, while the other method all became trapped in a local minimum.

**Table 3:** Registration results with an abrupt turn.

Method	Translation Error *e_t_*			Rotation Error *e_r_*		
	min	*μ_t_*	*σ_t_*	min	*μ_r_*	*σ_r_*
Slender bank experiment						
ICP	17.21 m	24.23 m	16.32 m	154.4°	176.1°	21.2°
M-ICP	13.23 m	21.23 m	18.31 m	156.6°	182.8°	33.9°
G-ICP	10.32 m	15.21 m	12.11 m	126.4°	192.09°	45.8°
OUR	0.31 m	4.55 m	3.98 m	0.1°	15.7°	8.2°
Triangular area experiment						
ICP	7.32 m	23.34 m	16.21 m	126.4°	189.1°	28.2°
M-ICP	16.42 m	26.57 m	15.31 m	145.6°	176.8°	35.9°
G-ICP	9.89 m	28.11 m	13.23 m	170.2°	181.9°	49.8°
OUR	0.21 m	6.78 m	4.98 m	0.4°	14.7°	7.4°

The rotation error along the *z*-axis was around 160°–220°.

### 4.5. Running Time

**Figure 11:**
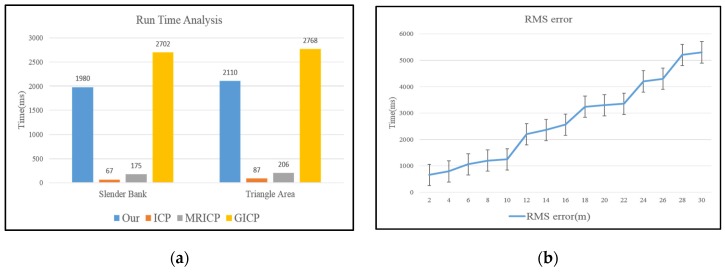
Time cost analysis: (**a**) in both Slender Bank test and Triangle Area test, our method could make the alignment around 2 s, faster than the generalized ICP, but slower than the standard-ICP method and multi-resolution ICP method because of the heuristic escape scheme; (**b**) the registration time is highly related to RMS error.

Figure 11a presents the average registration times of the methods in both experiments with normal initial transformation errors. The proposed method was not as fast as the single standard ICP or M-ICP method. This is because of the heuristic escape scheme, which combines potential direction estimation and G-ICP to escape from the local minimum. On average, our proposed method reduced the registration time by 30% compared to G-ICP while guaranteeing registration accuracy at the same time. For the abrupt turn case, we only evaluated the relation between the RMS error and registration time of our proposed method. As shown in Figure 11b, the registration time for the abrupt case was closely related to the heuristic escape time, as given in Figure 8. A larger initial transformation error increased the heuristic escape time, which also increased the computation time.

## 5. Conclusions

This paper presents the fast and accurate registration of a large-scale 3D environmental model for application to heterogeneous mobile robot cooperation with a rough initial transformation error. A hierarchical searching scheme is combined with the octree-based ICP algorithm. A novel early warning mechanism is proposed to perceive the local minimum problem, and a heuristic escape scheme is used to avoid local minima and achieve optimal registration. Experiments involving one UAV and one USV were conducted to verify the proposed technique, and the experimental results were compared with those of normal ICP registration algorithms. The results showed that the proposed algorithm is insensitive to the initial transformation error and can make effective heuristic escape decisions to resolve the local minimum problem.

## References

[1] Du M., Xing Y., Suo J., Liu B., Jia N., Huo R., Chen C., Liu Y. (2014). Real-time automatic hospital-wide surveillance of nosocomial infections and outbreaks in a large Chinese tertiary hospital. BMC Med. Inform. Decis. Mak..

[2] Zheng Y., Daniel E., Hunter A.A., Xiao R., Gao J., Li H., Maguire M.G., Brainard D.H., Gee J.C. (2014). Landmark matching based retinal image alignment by enforcing sparsity in correspondence matrix. Med. Image Anal..

[3] Handa A., Whelan T., McDonald J., Davison A.J. A Benchmark for RGB-D Visual Odometry, 3D Reconstruction and SLAM. Proceedings of the 2014 IEEE International Conference on Robotics and Automation.

[4] Salas-Moreno R.F., Glocken B., Kelly P.H.J., Davison A.J. Dense Planar SLAM. Proceedings of the IEEE International Symposium on Mixed and Augmented Reality (ISMAR).

[5] Jiang Y., Chen H., Xiong G., Scaramuzza D. ICP Stereo Visual Odometry for Wheeled Vehicles based on a 1DOF Motion Prior. Proceedings of the 2014 IEEE International Conference on Robotics and Automation.

[6] Nießner M., Dai A., Fisher M. Combining inertial navigation and ICP for real-time 3D surface reconstruction. http://graphics.stanford.edu/~niessner/papers/2014/0inertia/niessner2014combining.pdf.

[7] David G. (2004). Distinctive Image Features from Scale-Invariant Keypoints. Int. J. Comput. Vis..

[8] Smith S.M., Brady J.M. (1997). SUSAN—A new approach to low level image processing. Int. J. Comput. Vis..

[9] Johnson A. (1997). Spin-Images: A Representation for 3-D Surface Matching. Doctoral Dissertation.

[10] He Y., Mei Y. (2015). An efficient registration algorithm based on spin image for LiDAR 3D point cloud models. Neurocomputing.

[11] Dinh H.Q., Kropac S. Multi-resolution Spin-images. Proceedings of the IEEE Computer Society Conference on Computer Vision and Pattern Recognition.

[12] Assfalg J., Bertini M., Bimbo A.D., Pala P. (2007). Content-based retrieval of 3-D objects using spin image signatures. IEEE Trans. Multimed..

[13] Bao S.Y., Chandraker M., Lin Y., Savarese S. Dense Object Reconstruction with Semantic Priors. Proceedings of the IEEE Computer Society Conference on Computer Vision and Pattern Recognition.

[14] Ho H.T., Gibbins D. (2009). Curvature-based approach for multi-scale feature extraction from 3D meshes and unstructured point clouds. IET Comput. Vis..

[15] Besl P., McKay N. (1992). A method for registration of 3-D shapes. IEEE Trans. Pattern Anal. Mach. Intell..

[16] Chen Y., Medioni G. Object Modelling by Registration of Multiple Range Images. Proceedings of the 1991 IEEE International Conference on Robotics and Automation.

[17] Zhang Z. (1994). Iterative point matching for registration of free-form curves and surfaces. Int. J. Comput. Vis..

[18] Segal A., Haehnel D., Thrun S. Generalized-ICP. http://www.robots.ox.ac.uk/~avsegal/resources/papers/Generalized_ICP.pdf.

[19] Jost T., Hugli H. A Multi-resolution ICP with Heuristic Closest Point Search for Fast and Robust 3D Registration of Range Images. Proceedings of the Fourth International Conference on 3-D Digital Imaging Modeling.

[20] Jost T., Hugli H. A Multi-resolution Scheme ICP Algorithm for Fast Shape Registration. Proceedings of the First International Symposium on 3D Data Processing, Visualization and Transmission.

[21] Yang J., Li H., Jia Y. Go-ICP: Solving 3D Registration Efficiently and Globally Optimally. Proceedings of the 2013 IEEE International Conference on Computer Vision.

[22] Hornung A., Wurm K.M., Bennewitz M., Stachniss C., Burgard W. (2013). OctoMap: An efficient probabilistic 3D mapping framework based on octrees. Auton. Robot..

[23] Zeng M., Zhao F., Zheng J., Liu X. (2013). Octree-based fusion for real-time 3D reconstruction. Graph. Model..

[24] Rusu R.B., Blodow N., Beetz M. Fast Point Feature Histograms (FPFH) for 3D Registration. Proceedings of the 2009 IEEE International Conference on Robotics and Automation.

[25] Quigley M., Conley K., Gerkey B., Faust J., Foote T., Leibs J., Berger E., Wheeler R., Mg A. ROS: An Open-Source Robot Operating System. http://pub1.willowgarage.com/~konolige/cs225B/docs/quigley-icra2009-ros.pdf.

[26] Rusu R.B., Cousins S. 3D Is Here: Point Cloud Library (PCL). Proceedings of the IEEE International Conference on Robotics and Automation.

